# Si-Based Hydrogen-Producing Nanoagent Protects Fetuses From Miscarriage Caused by Mother-to-Child Transmission

**DOI:** 10.3389/fmedt.2021.665506

**Published:** 2021-05-13

**Authors:** Noriyoshi Usui, Shogo Togawa, Takuya Sumi, Yuki Kobayashi, Yoshihisa Koyama, Yukiko Nakamura, Makoto Kondo, Koh Shinoda, Hikaru Kobayashi, Shoichi Shimada

**Affiliations:** ^1^Department of Neuroscience and Cell Biology, Graduate School of Medicine, Osaka University, Suita, Japan; ^2^United Graduate School of Child Development, Osaka University, Suita, Japan; ^3^Global Center for Medical Engineering and Informatics, Osaka University, Suita, Japan; ^4^Addiction Research Unit, Osaka Psychiatric Research Center, Osaka Psychiatric Medical Center, Osaka, Japan; ^5^Division of Neuroanatomy, Department of Neuroscience, Yamaguchi University Graduate School of Medicine, Yamaguchi, Japan; ^6^Department of Cell Biology, Graduate School of Medicine, Osaka University, Suita, Japan; ^7^Institute of Scientific and Industrial Research, Osaka University, Ibaraki, Japan

**Keywords:** maternal-fetal infection, silicon, hydrogen, nanoagents, miscarriage, placenta, inflammation, oxidative stress

## Abstract

Mother-to-child transmission of viruses and bacteria increases the risk of miscarriage and various diseases in children. Such transmissions can result in infections and diseases in infants or the induction of an inflammatory immune response through the placenta. Recently, we developed a silicon (Si)-based hydrogen-producing nanoagent (Si-based agent) that continuously and effectively produces hydrogen in the body. Since medical hydrogen has antioxidative, anti-inflammatory, antiallergic, and antiapoptotic effects, we investigated the effects of our Si-based agent on mother-to-child transmission, with a focus on the rate of miscarriage. In pregnant mice fed a diet containing the Si-based agent, lipopolysaccharide (LPS)-induced miscarriage due to mother-to-child transmission was reduced and inflammation and neutrophil infiltration in the placenta were suppressed. We also found that the Si-based agent suppressed IL-6 expression in the placenta and induced the expression of antioxidant and antiapoptotic genes, such as *Hmox1* and *Ptgs2*. The observed anti-inflammatory effects of the Si-based agent suggest that it may be an effective preventative or therapeutic drug for miscarriage or threatened miscarriage during pregnancy by suppressing maternal inflammation caused by bacterial and viral infections.

## Introduction

Mother-to-child transmission occurs when a pathogen infecting the mother is transmitted to the child, and the infected child presents with symptoms (1, 2). It is associated with increased risks of miscarriage and various diseases in newborns. Mother-to-child transmission is classified into intrauterine infection during pregnancy, birth canal infection during childbirth, and breast milk infection after birth. Intrauterine infection is further divided into placental infection and ascending infection. Typical pathogens involved in transmission by each route are collectively referred to as TORCH pathogens, including *Toxoplasma*, others (HIV, measles virus, varicella virus, syphilis, and influenza virus), rubella virus, *Cytomegalovirus*, and herpes simplex virus (1–4). In recent years, neural progenitor cell death and microcephaly caused by Zika virus (5–7) and the unknown effects of SARS-CoV2 (8–10) have become serious problems during pregnancy. In addition, the maternal immune activation (MIA) caused by infections during pregnancy increases the risks of stillbirth and miscarriage as well as the risks of developmental disorders and psychiatric disorders in children (11, 12). Drug therapy is limited during pregnancy, and fetal treatment is rare, even if mother-to-child transmission is suspected, with most cases involving postnatal treatment. Thus, infections are difficult to treat during pregnancy, resulting in the development of various diseases in children, such as microcephaly, hydrocephalus, eye lesions, visual impairment, intrauterine growth retardation, epilepsy, psychomotor retardation, developmental disorders, and psychiatric disorders (3, 11, 12). Importantly, pregnancy is a critical period for fetal development. Damage during this stage has irreversible effects on all downstream processes in cell and tissue development. Therefore, the prophylactic or permanent treatment of mother-to-child transmission is preferable.

Medical hydrogen exerts antioxidative, anti-inflammatory, antiallergic, and antiapoptotic effects (13–16). Hydrogen selectively reduces hydroxyl radicals (•OH) in reactive oxygen species (ROS). Hydroxyl radicals effectively oxidize nucleic acids and lipids (17–19), thereby accelerating aging (20, 21) and contributing to oxidative stress-related diseases, such as cancer (22), diabetes (23), Parkinson's disease (24, 25), Alzheimer's disease (24, 25), and autism spectrum disorder (26). However, ROS other than hydroxyl radicals possess physiological functions; for example, hydrogen peroxide (H_2_O_2_) and superoxide anion radicals (^1^O_2_−) are utilized by the immune system. Therefore, it is important to eliminate only hydroxyl radicals without the removal of other ROS. Hydrogen reacts only with hydroxyl radicals and consequently, can be used as a therapeutic agent for diseases associated with oxidative stress, without side effects (27, 28). The preventive and therapeutic effects of hydrogen have been reported in many clinical and basic studies (29–31).

Hydrogen gas and hydrogen-rich water are used for the intake of hydrogen to remove hydroxyl radicals (32–34); however, this approach has various issues, such as accessibility, hydrogen volume, and intake. Even at a saturated hydrogen concentration (1.6 ppm), only 18 mL of hydrogen gas is included in 1 L of hydrogen-rich water. Moreover, the oral or intraperitoneal administration of saturated hydrogen-rich water transiently increases the hydrogen content in the blood, brain, and other organs throughout the body in rats, but the effect cannot be maintained for 60 min due to the permeability of hydrogen (35). To resolve such issues, we have developed a Si-based agent that can continually produce a large amount of hydrogen (up to 400 mL/g) by reaction with water under similar conditions (pH 8.3 and 36°C) to those in the gut (36–38). Unlike bulk Si, the Si nanostructures effectively react with water, resulting in hydrogen generation (39–42). However, previous studies of hydrogen generation using Si nanostructures have focused on its application to fuel cells and therefore use strong alkaline solutions to increase hydrogen generation rates. Si and its reaction product SiO_2_ are known to be non-toxic, enabling the oral administration of Si-based agents. We have reported the renoprotective and neuroprotective effects of enteric hydrogen generated from a Si-based agent in chronic kidney disease and Parkinson's disease model animals (38). Thus, a Si-based agent may resolve issues related to infection in pregnant women and fetuses via the continuous, efficient production of hydrogen in the body. Since hydrogen is produced in the gastrointestinal system, it is physically and easily delivered to the fetus.

In this study, we investigated the effects of our Si-based agent on mother-to-child transmission, particularly on rates of miscarriage. A mouse model of LPS-induced miscarriage caused by mother-to-child transmission was established (43, 44) and used to evaluate the effects of the Si-based agent in the diet (38). Our results demonstrate that Si-based agents act as anti-inflammatory and antioxidative agents to suppress miscarriage induced by maternal inflammation in mother-to-child transmission during pregnancy.

## Materials and Methods

### Mice

All procedures were performed according to the ARRIVE guidelines and relevant official guidelines under the approval (#27-010) of the Animal Research Committee of Osaka University. C57BL/6J (Japan SLC Inc., Shizuoka, Japan) pregnant female mice were used. For embryo staging, the day of detection of the vaginal plug was considered embryonic day (E) 0.5. Mice were housed in groups of 2–3 animals per cage (143 × 293 × 148 mm) in the barrier facilities of Osaka University under a 12 h light–dark cycle and given *ad libitum* access to water and food. Mouse body weights and food consumption were measured daily and at 16 and 20 h after LPS administration. The minimum number of animals for biological replicates was based on previous experiments to enable the detection of a significant difference between groups at *P* < 0.05. The initial average weights of pregnant mice did not differ significantly among groups. An experimenter blinded to the group setting performed all tests. The numbers (n) of animals used for each experiment are indicated in the appropriate figure legends. The preterm delivery rate was the ratio of the numbers of preterm delivery in each pregnant mouse calculated by the total number of litters. The survival rate was calculated based on the survival of the total number of litters in each group.

### LPS Administration

LPS was administered as described previously (45). Briefly, 1 mg/kg LPS (#L2880; Merck, Darmstadt, Germany) dissolved in saline (#3311401A2026; Otsuka Pharmaceutical Co., Ltd., Tokyo, Japan) was intraperitoneally injected at E15.5 and then at 16 h after the first administration. As reported in a previously published study (45), two times of 1 mg/kg LPS administration at 20 and 4 h prior to the endpoint did not cause maternal loss and induced associated fetal death and preterm birth. Another study has also reported that the administration of 1 mg/kg LPS did not affect the maternal survival rate (43). Mice in the control group were injected with saline intraperitoneally. Miscarriages were quantified 16 h after the first LPS administration and 4 h after the second LPS administration. Mice were euthanized with 5% isoflurane (#099-06571; FUJIFILM Wako Pure Chemical Corporation, Osaka, Japan) at 4 h after the second LPS administration, and samples were quickly obtained.

### Si-Based Agent and Treatment

The Si-based agent and Si-based agent-containing feed were prepared as described previously (38). A Si-based agent was produced from polycrystalline Si powder (Osaka Titanium Technologies Co., Ltd., Osaka, Japan; Si 4N). After milling the Si powder, surface treatment and aggregation were carried out. Therefore, the Si-based agent was composed of an aggregate of Si nanopowder. For the control laboratory chow, the AIN-93M diet (Oriental Yeast Co., Ltd., Tokyo, Japan) was used. For Si-based agent-containing laboratory chow, special laboratory chow was made containing 2.5 weight % Si-based agent in AIN-93M. The feed was given to pregnant mothers starting at E13.5, with free access to food and water. Before the animal experiments, hydrogen production from the feed and water was evaluated using a sensor gas chromatograph, SGHA-PA (FIS Inc., Hyogo, Japan).

### Hematoxylin–Eosin Staining

The mouse placenta was fixed with 4% PFA in water overnight at 4°C and then embedded in paraffin. Paraffin sections (7 μm thick) of the central (maximum diameter) region of the mouse placenta were deparaffinized and stained with Mayer's Hematoxylin Solution (#131-09665; FUJIFILM Wako Pure Chemical Corporation) and 1% Eosin Y Solution (#051-06515; FUJIFILM Wako Pure Chemical Corporation) according to the manufacturer's instructions. Cover glasses were mounted with Permount Mounting Medium (#SP15-100; Fisher Scientific, Pittsburgh, PA, USA) after dehydration. Images were collected using an all-in-one fluorescence microscope (BZ-X700; KEYENCE Corporation, Osaka, Japan).

### Immunohistochemistry

Immunohistochemistry was performed as described previously (46, 47). The mouse placenta was fixed with 4% PFA in PBS overnight at 4°C, cryoprotected in 30% sucrose in PBS overnight at 4°C, and then embedded in Tissue-Tek O.C.T. Compound (#4583; Sakura Finetek Japan Co., Ltd., Osaka, Japan) for cryosectioning. Cryosections (20 μm thick) of the central region of the mouse placenta were placed in PBS and then permeabilized in PBS-T for 30 min at room temperature. Blocking was performed using 10% goat serum and 1% BSA in PBS-T for 1 h at room temperature. Sections were stained with the following established primary antibodies overnight at 4°C: rabbit polyclonal anti-IL-6 (48) (1:200, #21865-1-AP; Proteintech, Rosemont, IL, USA) and rat monoclonal anti-Ly-6G (45) (1:200, #551459; BD Biosciences, San Jose, CA, USA). Sections were washed three times with PBS-T, incubated with species-specific antibodies conjugated to Alexa Fluor 488 and/or Alexa Fluor 597 (1:2,000; Invitrogen, Carlsbad, CA, USA) for 1 h at room temperature, and then washed 3 times with PBS-T. Cover glasses were mounted with Fluoromount/Plus (#K048; Diagnostic BioSystems, Pleasanton, CA, USA) or ProLong Diamond Antifade Mountant with DAPI (#P-36931 or #P36971, Thermo Fisher Scientific, Waltham, MA, USA) for nuclear staining. DAPI (#11034-56; Nacalai Tesque, Kyoto, Japan) was also used to stain nuclei. Antibody specificity was verified in antibody absorption experiments using recombinant proteins (data not shown). Images were collected using an Olympus microscope and digital camera system (BX53 and DP73; Olympus, Tokyo, Japan) and an all-in-one fluorescence microscope (BZ-X700, KEYENCE Corporation). Fluorescence intensities were quantified using KEYENCE analysis software with the Hybrid Cell Count application (KEYENCE Corporation).

### Quantitative Real-Time PCR

Quantitative real-time PCR (qPCR) was performed as described previously (49). Total RNA was extracted from the mouse placenta using the miRNeasy Mini Kit (#217004; Qiagen, Hilden, Germany) according to the manufacturer's instructions. Single-stranded cDNA was prepared using DNaseI, Amplification grade (#18068015; Thermo Fisher Scientific), and SuperScript III First-Strand Synthesis SuperMix (#18080400; Thermo Fisher Scientific) and amplified by PCR according to the manufacturer's instructions. qRT-PCR was performed using PowerUp SYBR Green Master Mix (#A25742; Thermo Fisher Scientific) and a QuantStudio 7 Flex Real-Time PCR System (Thermo Fisher Scientific). Each biological sample had four technical replicates for qPCR, and the number of biological replicates for each experiment is indicated in each figure legend. *18S* rRNA was used as a reference for normalization. Data were analyzed by the ΔΔCq method using QuantStudio 7 Flex Real-Time PCR System software (Thermo Fisher Scientific). The following primers were used: *18S rRNA*, F-5′-GAGGGAGCCTGAGAAACGG-3′, R-5′-GTCGGGAGTGGGTAATTTGC-3′; *Hmox1*, F-5′-GCCACCAAGGAGGTACACAT-3′, R-5′-CTTCCAGGGCCGTGTAGATA-3′; *Nqo1*, F-5′-GAAGCTGCA-3′, R-5′-GTTGTCGTACATGGCAGCAT-3′; *Cth*, F-5′-CTTGCTGCCACCATTACG-3′, R-5′-TTCAGATGCCACCCTCCT-3′; *Ptgs2*, F-5′-GCTTCGGGAGCACAACAG-3′; R-5′-TGGTTTGGAATAGTTGCTC-3′.

### Statistical Analysis

All data are presented as a means of biological independent experiments ± standard error of the mean (SEM). Statistical analyses (one-way ANOVA, two-way ANOVA, log-rank (Mantel-Cox) tests, unpaired *t*-tests, Mann–Whitney *U*-tests, and *F*-tests) were performed using Prism 7. *P* < 0.05 was considered to indicate statistical significance.

## Results

### Si-Based Agent Protected Against LPS-Induced Miscarriage in Mice

**Figure 1:**
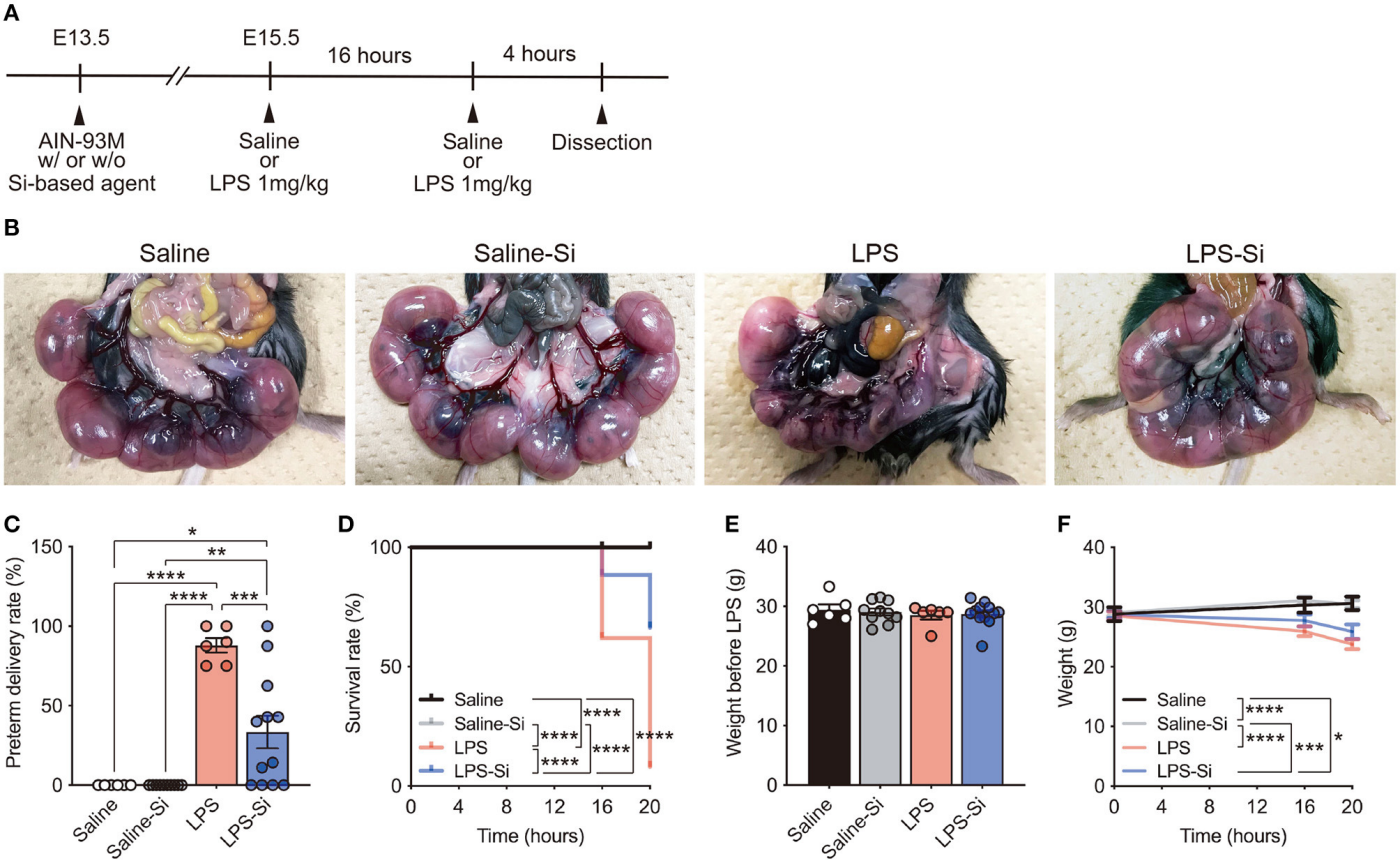
Silicon-based agent protects against miscarriage. **(A)** Experimental scheme. Pregnant mice were fed the AIN-93M diet with or without 2.5 weight % Si-based agent from E13.5 until dissection. Saline or lipopolysaccharide (LPS) (1 mg/kg) was intraperitoneally injected into pregnant mice at E15.5 and 16 h after the first administration. Quantification was performed at 16 and 20 h after the first administration. Pregnant mice were dissected at 4 h after the second administration. **(B)** Representative image showing the mouse uterus and fetus. The Si-based agent protects the fetus from LPS-induced miscarriage. **(C)** Preterm delivery rates (%) per litter for pregnant mice at 20 h after administration. **(D)** Survival rates (%) at 16 and 20 h after administration. The Si-based agent significantly improved the survival rate of fetuses. **(E)** Weight of pregnant mice before LPS-treatment. **(F)** Weight loss in LPS-treated pregnant mice at 16 and 20 h. Data are presented as means (± SEM). ****P < 0.0001, ***P < 0.001, **P < 0.01, *P < 0.05, two-way ANOVA or Log-rank (Mantel-Cox) test. The numbers of pregnant mice used for analyses of weight and preterm delivery rates were as follows: saline, *n* = 5; saline-Si, *n* = 10; LPS, *n* = 6; and LPS-Si, *n* = 12. The numbers of embryos used for survival rate analyses were as follows: saline, *n* = 90; saline-Si, *n* = 158; LPS, *n* = 50; and LPS-Si, *n* = 95 from 5 to 12 pregnant mice.

To investigate the effects of Si-based agents on miscarriage, we used a mouse model of LPS-induced miscarriage (44) (Figure 1A). Pregnant mice were fed the AIN-93M diet with or without 2.5 weight % Si-based agent from E13.5 until dissection. LPS (1 mg/kg) or saline was intraperitoneally injected into pregnant mice at E15.5, and the same treatments were administered for an additional 16 h (Figure 1A). No pregnant mice died after treatment with 1 mg/kg LPS, consistent with previous results (43, 45). We first quantified preterm delivery rates (%) per litter of pregnant mice at 20 h after the first administration (Figures 1B,C). A higher rate of preterm delivery per litter was observed in LPS-treated pregnant mice than in controls (Figure 1C). The LPS-induced increase in the preterm delivery rate was attenuated by the Si-based agent (Figure 1C). Furthermore, the survival rate (%) of fetuses was significantly lower in the LPS-treated mice than in controls. Again, the survival rate of LPS-treated pregnant mice improved significantly by treatment with the Si-based agent (Figure 1D). There was no difference in the weight of each group before LPS administration (Figure 1E). We found that the weights of pregnant mice at each time point were significantly lower in LPS-treated pregnant mice due to miscarriage (Figure 1F). We also found no negative effect of the Si-based agent on miscarriage and weight loss in the mice (Figures 1B–F). These results indicate that Si-based agents prophylactically and therapeutically protect the fetus from miscarriage induced by LPS.

### Protective Effect of the Si-Based Agent on the Placenta

**Figure 2:**
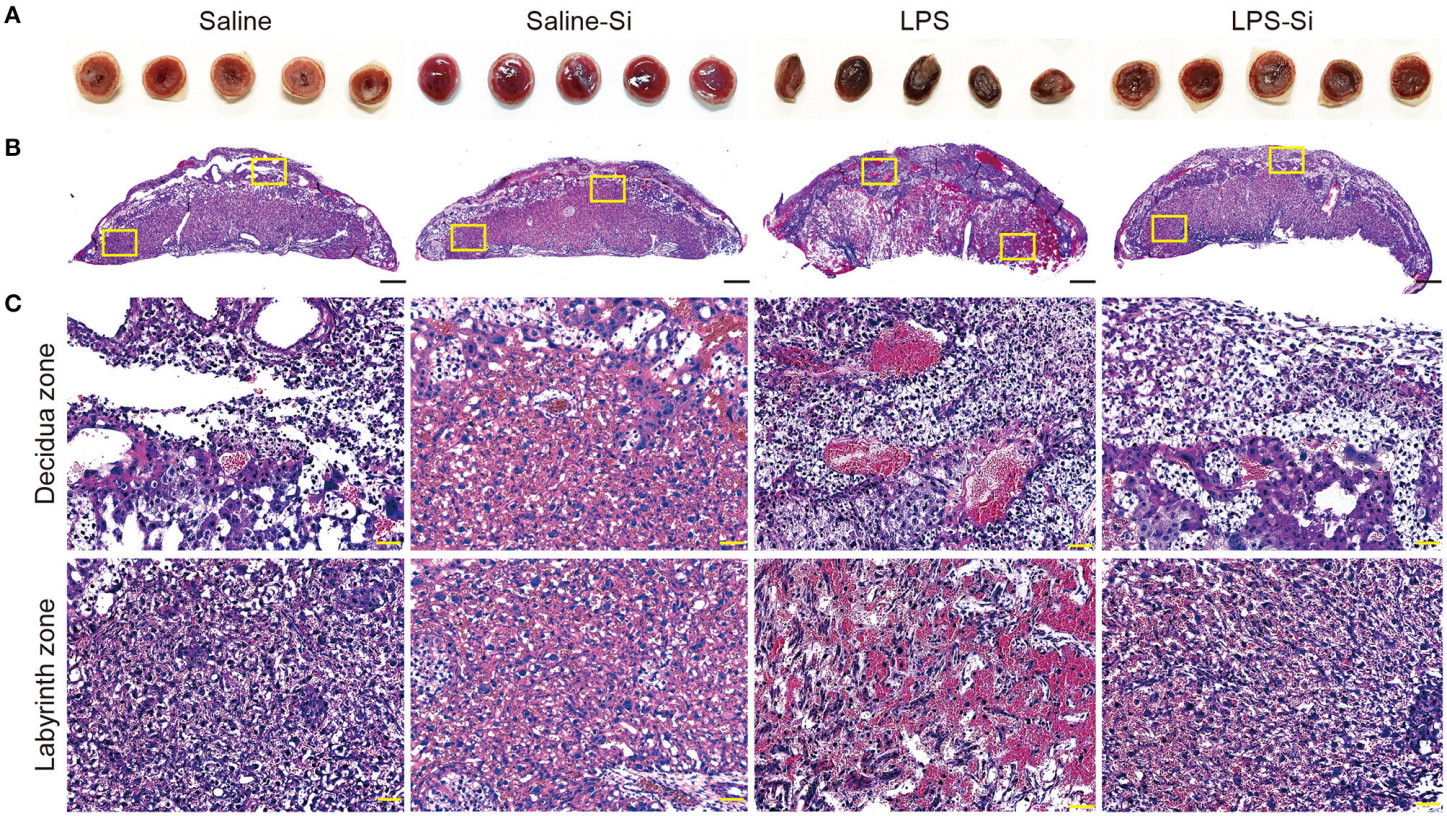
Protective effects of the Si-based agent on the placenta. **(A)** Representative image showing the central region of mouse placenta at 4 h after the second administration. The Si-based agent protected against placental atrophy. **(B)** Hematoxylin-eosin (HE) staining showing the mouse placental sections. **(C)** High-magnification images of mouse placental sections showing decidua and labyrinth zones highlighted in **(B)**. Neutrophil infiltration and vasodilation in the placenta were observed in LPS-treated mice, and placental damage due to inflammation was observed. Inflammation was attenuated by treatment with the Si-based agent. Scale bars: 500 μm in **(B)**, 50 μm in **(C)**.

We then analyzed the placenta 20 h after the first administration of LPS with or without the Si-based agent to examine the effect on inflammation. We observed several serial sections of the central (maximum diameter) region of the mouse placenta in each group. Placental atrophy and pigment changes were only observed in LPS-treated mice (Figure 2A). By HE staining of placental sections of LPS-treated mice, we observed the infiltration of inflammatory cells, such as neutrophils, as well as necrotic placentitis with necrosis, particularly in the labyrinth zone (Figures 2B,C).

### Anti-inflammatory Effect of the Si-Based Agent in the Placenta

**Figure 3:**
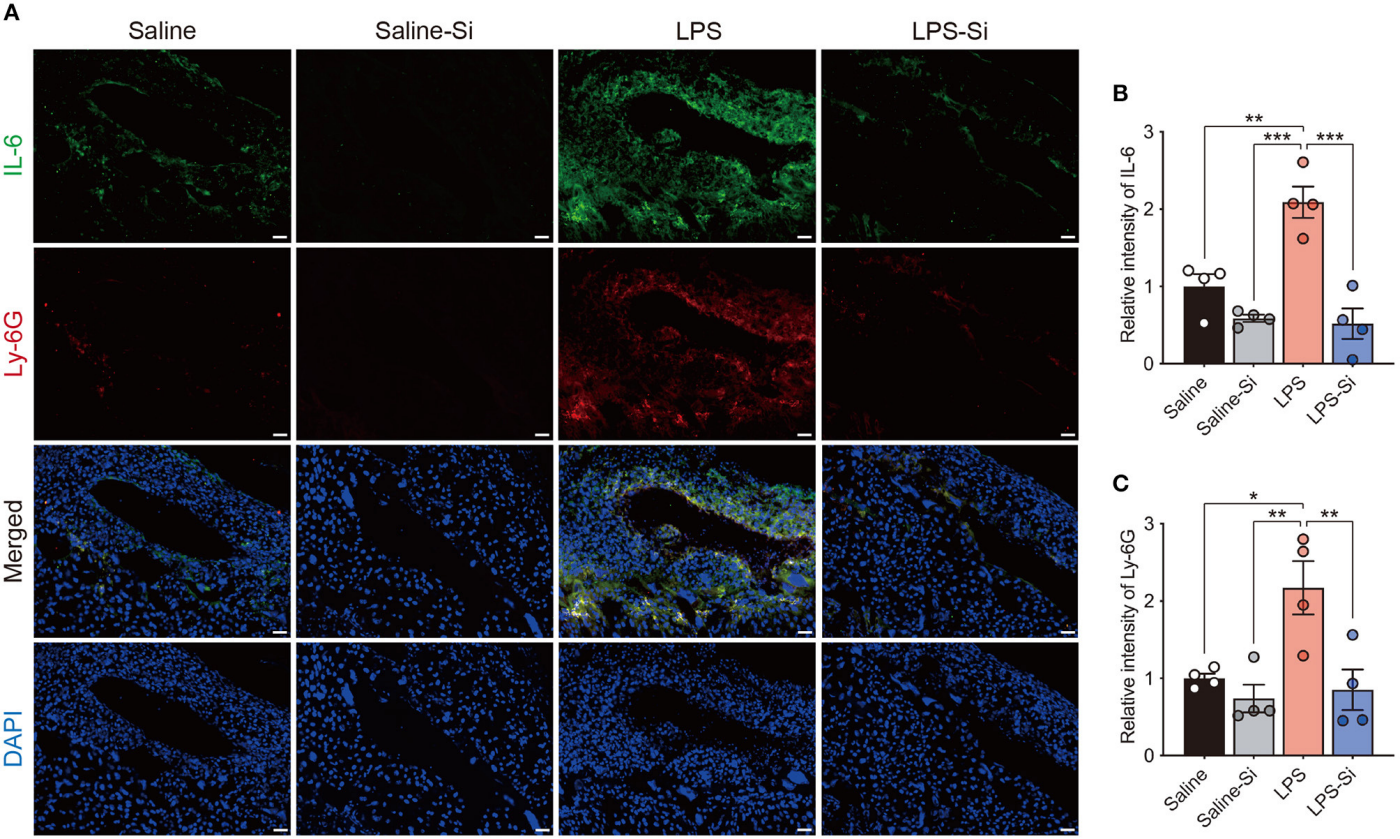
Anti-inflammatory effect of the Si-based agent in the placenta. **(A)** Fluorescent images of IL-6 and Ly-6G staining in the central region of mouse placenta at 4 h after the second administration. **(B,C)** Quantifications of relative intensities of IL-6 **(B)** and Ly-6G staining **(C)**. Placental inflammation and peripheral neutrophil aggregation induced by LPS were suppressed by the Si-based agent. *IL-6*: interleukin-6, a marker of inflammation*, Ly-6G:* a marker of peripheral neutrophils. Data are presented as means (± SEM). ***P < 0.001, **P < 0.01, *P < 0.05, one-way ANOVA. *n* = 4/condition. Scale bars: 50 μm.

To evaluate the effect of the Si-based agent on placental inflammation, we performed immunostaining of placental sections to detect the proinflammatory cytokine interleukin-6 (IL-6) (48) and the neutrophil marker Ly-6G (45). Strong merged signals of IL-6 and Ly-6G were observed in the placenta of LPS-treated mice (Figure 3A). In quantitative analyses, we found significantly higher intensities of both IL-6 and Ly-6G signals in the placentas of LPS-treated mice than in control mice (Figures 3B,C). In contrast, the intensities of IL-6 and Ly-6G signals in LPS-treated mice decreased significantly by treatment with the Si-based agent to the levels observed in the control (Figures 3B,C). These results indicate that the Si-based agent has an anti-inflammatory effect against LPS-induced inflammation. These results also suggest that the Si-based agents suppress the maternal inflammation caused by bacterial and viral infections.

### Antioxidant and Antiapoptotic Genes Contribute to the Anti-inflammatory Effect of the Si-Based Agent

**Figure 4:**
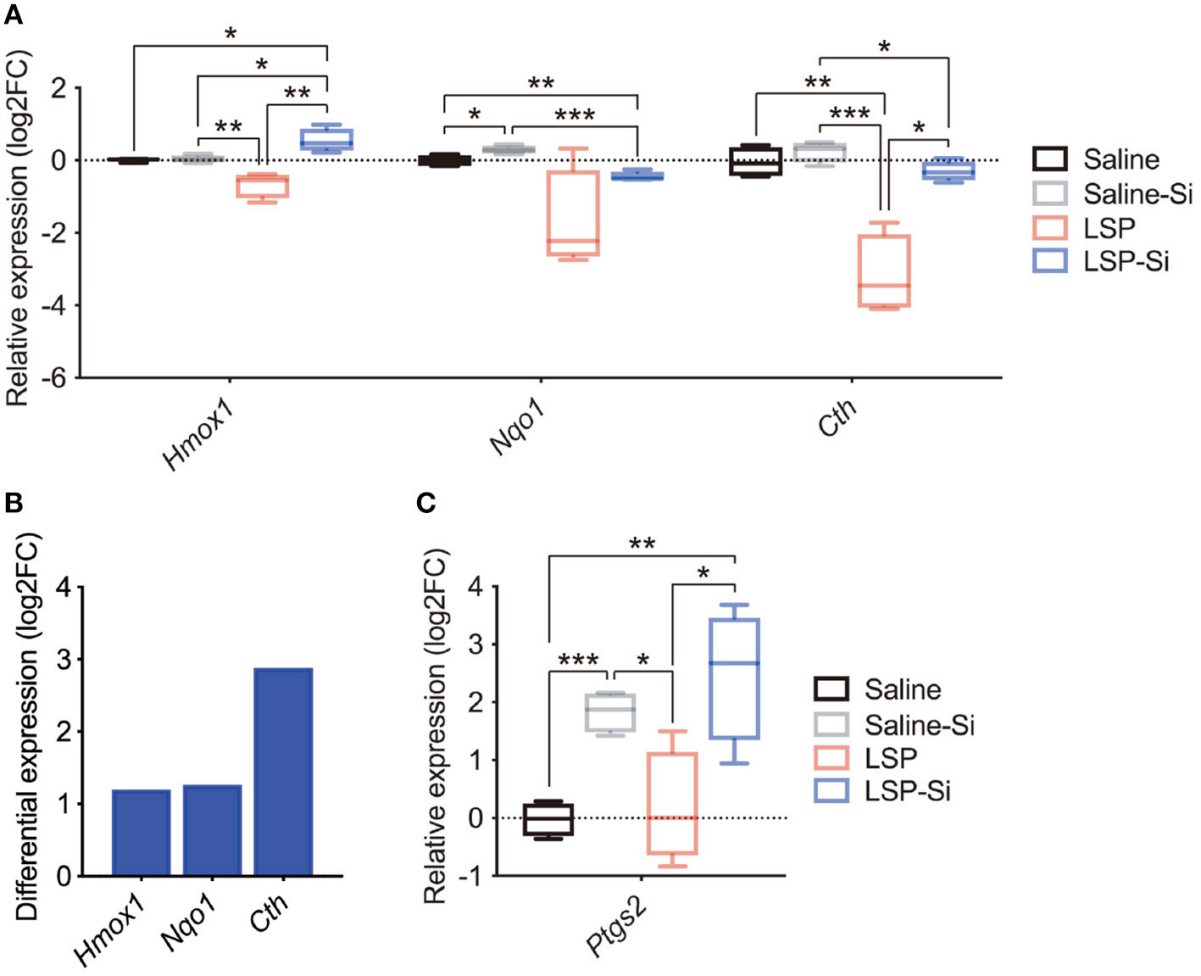
Expression levels of antioxidant and antiapoptotic genes after treatment with the Si-based agent. **(A)** Relative expression levels of antioxidant genes (*Hmox1, Nqo1*, and *Cth*) in the whole mouse placenta at 4 h after the second administration. *Hmox1* expression was significantly elevated in the placenta of LPS-treated mice administered the Si-based agent. **(B)** Differential expression analysis of the effect of Si-based agent in LPS-treated mice. **(C)** Relative expression levels of the antiapoptotic gene *Ptgs2* in the mouse placenta at 4 h after the second administration. Significant increases in the expression of *Ptgs2* were observed in the placentas of Si-based agent-treated mice and LPS-treated mice administered the Si-based agent. Data are presented as means (±SEM). Asterisks indicate ***P < 0.001, **P < 0.01, *P < 0.05, unpaired *t*-test or Mann–Whitney *U*-test. *n* = 4/condition.

Si-based agents produce hydrogen and have antioxidative effects (38). A recent study also reported that antioxidant genes, such as *Hmox1* and *Nqo1*, play a role in the protective effects against inflammation (50–55). Thus, we examined the expression of antioxidant genes (*Hmox1, Nqo1*, and *Cth*) in the placenta by qPCR to further investigate the effects of the Si-based agent. The expression levels of antioxidant genes were lower in the placentas of LPS-treated mice than in control mice (Figure 4A). The expression levels of antioxidant genes in mice treated with both the Si-based agent and LPS were similar to those in the control group (Figure 4A). In particular, *Hmox1* levels were significantly higher in mice treated with the Si-based agent and LPS than in control mice and in mice treated with LPS alone (Figure 4A). We detected high expression levels of antioxidant genes in LPS-treated mice administered the Si-based agent than in those without the Si-based agent (Figure 4B). We also found that the Si-based agent did not affect those gene expressions at the basal level, except *Nqo1* (Figure 4A). We further examined the expression of the antiapoptotic gene *Ptgs2* in the placenta. *Ptgs2* levels were significantly higher in Si-based agent-treated mice and Si-based agent-treated LPS-treated mice than in the other groups (Figure 4C). These results demonstrate that the anti-inflammatory effects of the Si-based agent are mediated by antioxidant and antiapoptotic genes.

## Discussion

This study demonstrated that a Si-based agent prophylactically and therapeutically acts as an anti-inflammatory agent to suppress miscarriage induced by maternal inflammation during pregnancy. The Si-based agent rescued placental impairment by LPS-induced inflammation and neutrophil infiltration. Si-based agents increased the expression of antioxidant and antiapoptotic genes in the placenta, even in mice administered high-dose LPS. The induction of antioxidant and antiapoptotic gene expression by Si-based agents may contribute to these protective effects. Our results demonstrate that Si-based agents prophylactically and therapeutically function in both inflammation and oxidative stress.

Previous studies of medical hydrogen have reported that hydrogen-rich water and medical hydrogen gas exert antioxidative, anti-inflammatory, antiallergic, and antiapoptotic effects (13–16). Medical hydrogen also effectively reduces ROS in redox reactions (56). There are several possible explanations for the inhibitory effects of the Si-based agent on miscarriage, including the suppression of (1) inflammation or oxidative stress itself, (2) inflammation mediated by LPS-induced oxidative stress, and (3) oxidative stress mediated by LPS-induced inflammation.

LPS induces inflammation by the activation of IL-6 via TLR4 downstream signaling. In LPS-treated mice, we observed significantly increased levels of IL-6 and neutrophil infiltration in the placenta, disrupting placental function (Figures 2, 3). In such conditions, the normal function of the placental barrier between the mother and fetus may not be maintained. Previous studies have reported that IL-6 levels are elevated in the amniotic fluid, umbilical cord plasma, and neonatal plasma in preterm delivery and miscarriage (57–61). Maternal IL-6 is induced by infections, such as TORCH pathogen infections (1–4). Therefore, in mother-to-child transmission, IL-6 is a key factor in preterm delivery and miscarriage. Maternal hyperinflammation causes miscarriage and damages the fetus directly through the placenta.

In redox reactions, ROS and free radicals are produced at the site of inflammation and are deeply involved in the inflammatory response, while interacting with other mediators and cells (13–16). In particular, hydroxyl radicals are extremely reactive and oxidize biological components, such as nucleic acids, proteins, lipids, and carbohydrates (17–19). ROS and free radicals are produced by neutrophils, macrophages, intracellular mitochondria, and the arachidonic acid cascade of the vascular endothelium that infiltrates inflammatory foci (62–64). Our results suggest that hydrogen produced by a Si-based agent may suppress the onset and spread of inflammation by acting directly on ROS and free radical production during inflammation.

The LPS-induced activation of ROS and oxidative stress via downstream TRIF and TLR4 signaling (65, 66) further leads to the activation of NF-κB signaling and eventually to the production of proinflammatory cytokines, including IL-6, and inflammation (65, 67). In other words, LPS administration causes both inflammation and oxidative stress, and these effects may be attenuated by Si-based agents. We found that the expression levels of antioxidant genes (*Hmox1, Nqo1*, and *Cth*) were maintained in the placenta of LPS-treated mice after the administration of the Si-based agent (Figure 4). We also found significantly higher expression levels of the antioxidant gene *Hmox1* in LPS-treated mice administered the Si-based agent than in other mice (Figure 4). *Hmox1* and *Nqo1* play central roles in the redox reaction via NFE2L2 (50–55). Under normal conditions, NFE2L2 forms a dimer with KEAP1, and NFE2L2 is ubiquitinated and degraded by the proteasome (50–54). However, under oxidative stress, NFE2L2 is released from KEAP1 and translocates into the nucleus to turn on the expression of antioxidant genes, such as *Hmox1* and *Nqo1* (50–55). KEAP1 senses oxidative stress directly through multiple cysteine residues (68). HMOX1 and NQO1, downstream factors of NFE2L2, suppress TNFα, IL-1β, and IL-6 production by suppressing the effects of hydrogen peroxide (55, 65, 69).

Our results suggest that Si-based agents activate the redox reaction of the NFE2L2 pathway, such as the downstream factor HMOX1. However, the molecular mechanisms by which our Si-based agent directly and constantly activate the pathway are unknown. Typically, a non-lethal amount of LPS induces *Hmox1* and *Nqo1* expression at the mRNA and protein levels in cultured human monocytes (69). In other words, LPS induces the expression of these genes within the range of non-lethal doses. The high-dose administration of cytotoxic mycotoxins decreases HMOX1 expression in cultured rat kidney cells (70). Thus, in the placenta, which is severely damaged by inflammation, antioxidant genes might not be effectively induced. In addition, viruses, such as hepatitis C virus (HCV) (71) and spring viremia of carp virus (SVCV) (72), specifically downregulate HMOX1 during infection. In the case of HCV, the expression levels of HMOX1 and *Hmox1* mRNA in the HCV-infected liver were more than 4-fold lower than those in controls, with no differences in superoxide dismutase (SOD) expression (71). Si-based agents may prevent the disruption of the intrinsic defense system caused by such pathogenic infections. *Ptgs2* is an antiapoptotic gene that plays a role in protection against apoptosis, cell proliferation, angiogenesis, and fatty acid metabolism (73). In this study, we found significantly and remarkably increased expression levels of *Ptgs2* in the placenta of LPS-treated mice administered the Si-based agent than in other mice (Figure 4), suggesting that the Si-based agent activates antiapoptotic signaling.

Collectively, Si-based agents exert anti-inflammatory, antioxidative, and antiapoptotic effects. However, the detailed molecular mechanism underlying the effect of Si-based agents needs to be clarified in future research. In addition to the LPS-treated model, it is necessary to consider an infection model, such as models of *Toxoplasma* or influenza virus infections, to evaluate the effects of Si-based agents on mother-to-child transmission. Eventually, the safety of SI-based agents for pregnant mothers and fetuses needs to be proven.

It has recently been reported that after abnormal increases due to infections and drug administration, blood cytokines spread throughout the entire body and that a cytokine storm is generated by positive feedback among various cytokines and leukocytes (61, 74). In the cytokine storm, neutrophil activation and vasodilation cause organ failure and even death (61, 74). Cytokine storms during pregnancy might result in fetal lethality, preterm delivery, and miscarriage (75). In this study, a high dose of LPS was used to clearly establish the effects of the Si-based agent (43, 44), demonstrating a significant suppressive effect on miscarriage and *Hmox1* expression. Our results suggest that our Si-based agent may also be effective against infections as well as cytokine storms and other inflammatory diseases.

This study has limitations. The Si-based agent was given in the diet before LPS administration. Although it was effective as a therapeutic drug after LPS administration, the results of this study cannot be ruled out as having a strong prophylactic aspect. In addition, although mice can freely access Si-based agents at any time, the amount of food consumed by each mouse was not quantified. However, our Si-based agent was given in sufficient amounts in advance equally (data not shown), suggesting that it continuously produces hydrogen in the mouse gut.

Taken together, our Si-based agent prophylactically and therapeutically functions as an anti-inflammatory, antioxidative, and antiapoptotic agent in LPS-induced miscarriage model animals. Our results suggest that a Si-based agent may act as a preventative drug for miscarriage or a therapeutic drug for threatened miscarriage during pregnancy by suppressing maternal inflammation, oxidative stress, and placental dysfunction caused by bacterial and viral infections.

## Data Availability Statement

The original contributions presented in the study are included in the article/supplementary material, further inquiries can be directed to the corresponding author/s.

## Ethics Statement

The animal study was reviewed and approved by Animal Research Committee of Osaka University.

## Author Contributions

NU designed the study, analyzed the data, and wrote the paper. NU, ST, TS, YN, and MK performed experiments and quantitative analyses. YuK, YoK, and HK developed a method for the fabrication of Si-based agents. KS and SS supervised this study and provided intellectual guidance. All authors discussed the results and commented on the manuscript.

## Conflict of Interest

The authors declare that the research was conducted in the absence of any commercial or financial relationships that could be construed as a potential conflict of interest.
